# Large-scale decontamination of disposable FFP2 and FFP3 respirators by hydrogen peroxide vapour, Finland, April to June 2020

**DOI:** 10.2807/1560-7917.ES.2022.27.11.2100119

**Published:** 2022-03-17

**Authors:** Katri Laatikainen, Markku Mesilaakso, Ilpo Kulmala, Erja Mäkelä, Petri Ruutu, Outi Lyytikäinen, Susanna Tella, Tarmo Humppi, Satu Salo, Tuuli Haataja, Kristiina Helminen, Henri Karppinen, Heli Kähkönen, Tarja Vainiola, Kirsimarja Blomqvist, Sirpa Laitinen, Kati Peltonen, Marko Laaksonen, Timo Ristimäki, Jouni Koivisto

**Affiliations:** 1 Department of Separation Science, Lappeenranta-Lahti University of Technology LUT, Lappeenranta, Finland; 2 LAB University of Applied Science, Lappeenranta and Lahti, Finland; 3 Finnish Defense Research Agency, Ylöjärvi, Finland; 4 VTT Technical Research Centre of Finland Ltd, Espoo and Tampere, Finland; 5 Finnish Institute of Occupational Health, Helsinki, Kuopio and Oulu, Finland; 6 Finnish Institute for Health and Welfare, Helsinki, Finland; 7 Finnish Medicines Agency Fimea, Helsinki, Finland; 8 LUT School of Business and Management, Lappeenranta-Lahti University of Technology LUT, Lappeenranta, Finland

**Keywords:** FFP2/FFP3 respirators, H_2_O_2_ decontamination, reuse, multidisciplinary, multisectoral collaboration

## Abstract

**Background:**

The shortage of FFP2 and FFP3 respirators posed a serious threat to the operation of the healthcare system at the onset of the COVID-19 pandemic.

**Aim:**

Our aim was to develop and validate a large-scale facility that uses hydrogen peroxide vapour for the decontamination of used respirators.

**Methods:**

A multidisciplinary and multisectoral ad hoc group of experts representing various organisations was assembled to implement the collection and transport of used FFP2 and FFP3 respirators from hospitals covering 86% of the Finnish population. A large-scale decontamination facility using hydrogen peroxide vapour was designed and constructed. Microbiological tests were used to confirm efficacy of hydrogen peroxide vapour decontamination together with a test to assess the effect of decontamination on the filtering efficacy and fit of respirators. Bacterial and fungal growth in stored respirators was determined by standard methods.

**Results:**

Large-scale hydrogen peroxide vapour decontamination of a range of FFP2 and FFP3 respirator models effectively reduced the recovery of biological indicators: *Geobacillus stearothermophilus* and *Bacillus atrophaeus* spores, as well as model virus bacteriophage MS2. The filtering efficacy and facial fit after hydrogen peroxide vapour decontamination were not affected by the process. Microbial growth in the hydrogen peroxide vapour-treated respirators indicated appropriate microbial cleanliness.

**Conclusions:**

Large-scale hydrogen peroxide vapour decontamination was validated. After effective decontamination, no significant changes in the key properties of the respirators were detected. European Union regulations should incorporate a facilitated pathway to allow reuse of appropriately decontaminated respirators in a severe pandemic when unused respirators are not available.

## Introduction

The coronavirus disease (COVID-19) pandemic has led to a high demand for personal protective equipment (PPE). The World Health Organization (WHO) recommends the use of filtering facepiece (FFP2 and FFP3) respirators or equivalent for healthcare workers caring for COVID-19 patients when performing aerosol-generating procedures [1]. In view of delays in scaling up production, since manufacturing respirators is technically demanding, the use of decontaminated respirators has been suggested as a solution to ensure appropriate protection for healthcare workers if new respirators are not available. European Union (EU) regulation prevents the use of decontaminated respirators if the respirator does not have instructions for decontamination from the manufacturer [2]. The United States (US) Food and Drug Administration (FDA) has given Emergency Use Authorization for reuse of decontaminated respirators [3].

Hydrogen peroxide (H_2_O_2_) vapour (HPV) has been used for surface decontamination in hospitals and biological laboratories [4] because of its wide spectrum of antimicrobial activity, good penetration ability, material compatibility and absence of harmful residues [5,6].

Decontamination of respirators using five methods was compared by Viscusi et al. in 2009 [7]: (i) ultraviolet radiation (UV); (ii) ethylene oxide (EtO) treatment; (iii) HPV treatment; (iv) microwave treatment; and (v) hypochlorite treatment. Subsequently, laboratory-scale investigations [8,9] and literature reviews [6,10-14] have found HPV to be one of the most suitable methods for decontaminating respirators.

Scaling up the decontamination with HPV of large numbers of used respirators or other decontamination agents has not previously been reported. We describe how a large-scale facility for decontamination of a range of used FFP2 and FFP3 respirator models collected from nine Finnish hospitals using HPV was developed and validated during a short period of time in spring 2020.

## Methods

A multidisciplinary and multisectoral ad hoc group of experts representing various organisations was assembled to implement the collection and transport of used respirators from April to June 2020. The group consisted of the Lappeenranta-Lahti University of Technology LUT, the LAB University of Applied Science, the Finnish Defence Research Agency (FDRA, the VTT Technical Research Centre of Finland Ltd (VTT), the Finnish Institute of Occupational Health, the Finnish Institute for Health and Welfare and the Finnish Medicines Agency Fimea.

### Collection of respirators

Nine of 20 invited healthcare districts (HD), covering ca 86% of the Finnish population, joined the project. The project group developed guidelines and coordinated and organised the local collection and storage (at +2 to + 6 °C) of used respirators together with infection control teams and logistics management experts in the HD. Transportation from participating hospitals to the HPV decontamination facility was organised by the FDRA/Finnish Defence Forces (FDF), with distances ranging between 30 km and 600 km.

### Design of the decontamination facility

**Figure 1:**
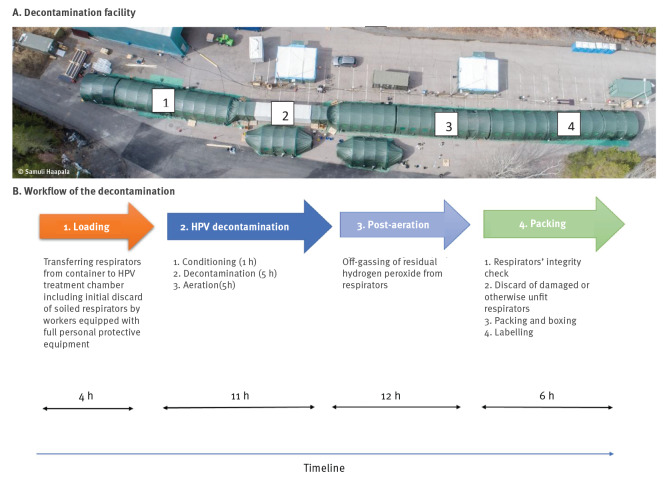
Aerial view of the decontamination facility (A) and workflow of the decontamination of respirators using hydrogen peroxide vapour (B), Finland, April–June 2020

The FDRA and FDF, in collaboration with VTT, designed and constructed the decontamination facility for respirators at FDRA’s premises (Figure 1).

HPV: hydrogen peroxide vapour.

The facility was built on the Finnish Defence Research Agency’s premises. The timeline provided is for the decontamination of 20,000 respirators.

Photo: Finnish Defence Forces/Samuli Haapala.

The HPV decontamination chamber consisted of a 12 m long thermally isolated standard container, modified by constructing an 8 m long, 47 m^3^ inner chamber, with stainless steel and aluminium inner lining. The chamber was equipped with a heating system, fans and an air mixing unit to distribute airflows vertically and horizontally. A Cleamix VCS-100Cr series generator (Cleamix, Toivala, Finland) [15,16] with one or two additional parallel units used 50% w/w H_2_O_2_ solution to generate HPV. After HPV treatment, the chamber was flushed with high-efficiency particulate arrestance (HEPA)-filtered supply air (1,000 m^3^/ h) to quickly bring down the H_2_O_2_ concentration. Exhaust air was discharged through an activated carbon filter to minimise the dispersion of HPV outside the decontamination chamber. In total, 13 HPV exposure treatment runs were carried out during the study.

The generators in the HPV decontamination chamber had H_2_O_2_ sensors Peroxcab HP 272 (Vaisala, Helsinki, Finland) which measured air temperature, humidity and H_2_O_2_ concentrations and guided the control of the decontamination process. Two chemical indicators, 3M 1228 indicator tape (3M, St Paul, US) and Steraffirm VH2O2 process indicator (Steris Life Sciences, Mentor, US) were used in parallel to verify that adequate H_2_O_2_ concentrations were reached. The total HPV exposure cycle threshold (Ct) value was calculated as concentration (in parts per million (ppm))*time (in hours).

### Biological indicators of decontamination efficacy

To confirm the efficacy of decontamination, we employed two different indicators consisting of bacterial spores, as well as a model virus indicator. Each Spordex VH2O2 biological indicator (Steris Life Sciences) contained 2.0 x 10^6^ *Geobacillus stearothermophilus* spores inoculated on a stainless steel matrix. The manufacturer’s instructions were followed to culture and interpret the indicator. A pass result indicated a ≥ 6 log_10_ reduction in microbial viability.

Our in-house biological indicator consisted of *Bacillus atrophaeus* (BG) spores (VTT E-052737, VTT Culture Collection). On each 1 x 1 cm test piece cut from FFP3 type 3M Aura 9332 + or FFP2 type 3M Aura 06923 + respirators, 0.1 ml of BG suspension with a concentration of 9 log_10_ colony-forming units (cfu)/mL spores was applied, together with Tween 20 to improve penetration into the respirator matrix. The control and decontaminated test pieces were shaken in physiological saline containing peptone medium and cultured following standard methods before counting colonies.

The MS2 bacteriophage (DSM 13767) was used as a model virus decontamination indicator. In brief, 0.1 ml of a suspension containing 1.4 x 10^7^ phages/mL was applied on 1 x 1 cm test pieces of respirators. The MS2 virus was enumerated with *Escherichia coli* VTT E-113164 on a Nutrient Agar plate (37 °C, 1 day).

The reduction in microbial viability for BG and MS2 biological indicators was defined as the relative number of microbes cultured from non-decontaminated vs decontaminated test pieces and expressed as log_10_ reduction in microbial recovery.

### Assessing contamination during storage

In order to assess bacterial or fungal contamination during storage before and after HPV treatment, used respirators were tested according to the EN 14683 standard [17]. The respirator was placed in a bottle containing 300 ml of extraction liquid (1 g/L peptone, 5 g/L NaCl and 2 g/L Tween 20) and 100 ml aliquots were filtered through 0.45 μm filters and cultured for the total viable aerobic microbial count, as well as for fungi using selective media.

### Assessing filtering efficacy and breathing resistance

**Figure 2:**
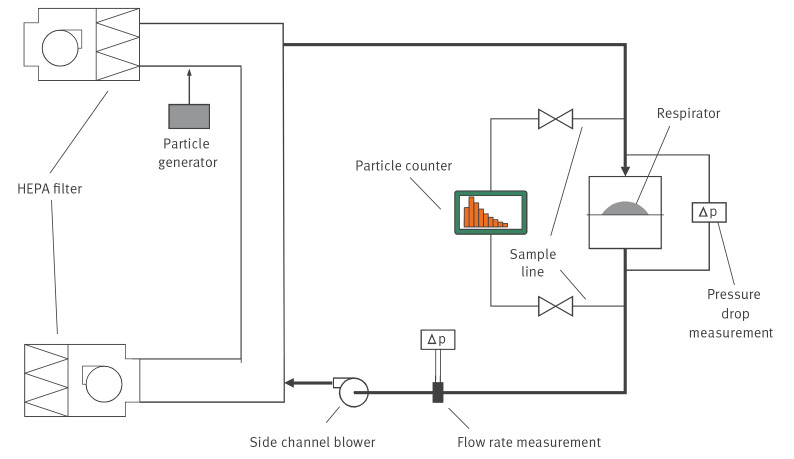
Respirator filtration efficiency and breathing resistance measurement system, Finland, April–June 2020

Filtering efficacy and breathing resistance of respirators were determined using the measurement system shown in Figure 2. Diethylhexyl sebacate (DEHS) particles were fed into a HEPA-filtered air stream, a fraction of which was diverted to the respirator. The airflow through the respirator was created using a side channel blower and the flow rate was measured with an orifice plate. The concentration of DEHS particles upstream and downstream of the respirator were measured with a Palas Fidas Frog optical particle counter (Palas GmbH, Karlsruhe, Germany) in the size range of 0.18–1.5 µm. The respirators were tested at an airflow of 90 L per minute, as defined by the EN 149 standard [18].

Used and decontaminated respirators were randomly selected to test for filtering efficacy and breathing resistance. These included 132 FFP2 respirators from two different manufacturers and 133 FFP3 respirators from six different manufacturers, including respirators with and without valves (Table 1).

**Table 1:** FFP2 and FFP3 respirators tested for filtering efficacy and breathing resistance after decontamination with hydrogen peroxide vapour, Finland, April–June 2020 (n = 265)

Respirators tested	n
FFP2 respirators	
3M 9322	10
3M Aura 06923 +	62
3M Aura 1872V +	5
3M Aura 9322 +	44
3M K112	10
Medline None 24508	1
**Total**	**132**
FFP3 respirators	
3M 1873V	10
3M 8835 +	4
3M Aura 1863 +	2
3M Aura 1873V +	6
3M Aura 1883 +	40
3M Aura 8835 +	2
3M Aura 9332 +	44
Climax 1730	6
JSP springfit 435 ML	2
Segre CN P3 V	15
UVEX silv-air 2310	1
Valmy VRV303	1
**Total**	**133**

FFP2/FFP3: filtering facepiece respirator.

### Testing the fit of respirators

Fit testing to ensure that respirators remained protective for individual users [19] was carried out using a condensation particle counter, the TSI PortaCount respirator fit tester 8048 (TSI Inc, Shoreview, US), before and after 10 and 20 HPV treatment cycles. In these separate experiments, the HPV treatment cycles of the respirators for fit testing were carried out in a smaller chamber, with the same exposure times and concentrations of HPV as used in the large decontamination facility. The tests were carried out according to the PortaCount manual, e.g. the N95-Companion option was used for class FFP2 respirator testing. Ten respirators from each of six types of respirators were tested (3M: K113, 1833 + , K112, 9322, 9322 + ; GlaxoSmithKline: Antiviral Respirator Mask). During testing, particle concentration was measured both inside the respirator and from the breathing zone outside the respirator. The quotient of the two concentrations was called the protection factor. The test result, or over-all fit factor, was the harmonised mean of the fit factors in each test section. An over-all fit factor of 100 was used as the threshold criterion for a pass result. PortaCount fit testing has been proposed as the inward leakage test method of choice for respirators certified by the National Institute of Occupational Safety and Health [20].

## Results

It took 12 weeks to progress from the first coordination meeting of the multisectoral group to the HPV decontamination run with the maximum load of 20,000 respirators. This period included the construction of the HPV decontamination facility, optimisation of decontamination conditions and implementation of quality assurance processes.

Approximately 45,000 respirators were subjected to decontamination. They were systematically checked for visible smearing, intactness of rubber gaskets and by distending the elastic straps after decontamination. Initially, ca 75% of the respirators were discarded at the decontamination facility, mainly because of smearing by cosmetic products. After an intensive information campaign to avoid use of cosmetics (e.g. make-up), the proportion discarded decreased to less than half of respirators collected.

The total HPV exposure Ct value in the decontamination runs ranged between 900 and 1,800 ppm*hours. The pairs of chemical indicators in the chamber, the number of which varied from five to 18 depending on respirator load, confirmed achievement of the target HPV exposure for 99.4% (159/160) of the indicator pairs used across the 13 treatment runs. The duration of HPV treatment varied between 1.5 h and 5.5 h depending on the number of respirators, but loading, airing and unloading the chamber approximately doubled the total duration of each run. The largest decontamination run lasted 11 h and contained 20,000 respirators. Airing of the decontaminated respirators continued outside of the chamber for up to 24 h.

### Biological indicators of decontamination efficacy

Of the commercial Spordex biological indicators, which were not available for the two first runs, 149/150 (99.3%) gave a pass result, indicating ≥ 6 log_10_ reduction in the viability of the *G. stearothermophilus* spores. In one decontamination run, one of 11 Spordex indicators did not show a pass result, but all 11 in-house BG spore indicators indicated ≥ 6 log_10_ reduction in spore viability.

**Figure 3:**
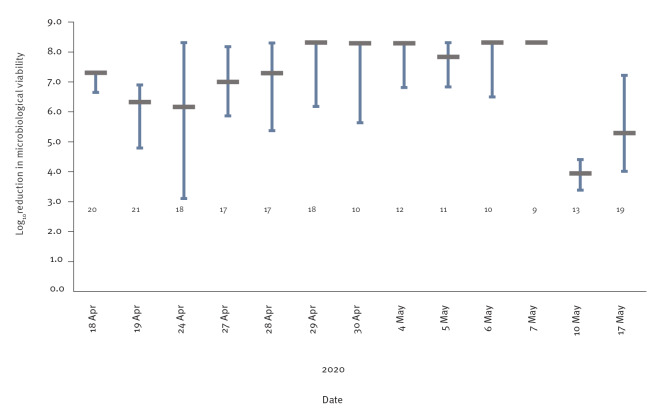
Reduction of the viability of *Bacillus atrophaeus* spores in 13 decontamination runs with hydrogen peroxide vapour, Finland, April–June 2020

The average growth of BG spores in the in-house biological indicator control test pieces ranged between 7.2 and 8.3 log_10._ In decontamination runs conducted between 18 April and 5 May 2020 with up to 2,760 respirators in each run, the majority of indicator test pieces demonstrated > 6 log_10_ reduction in BG viability compared with controls (Figure 3). In the first three decontamination runs, 43/58 (74.1%) respirator test pieces indicated ≥ 6 log_10_ reduction in BG viability. In the remaining test pieces of these three decontamination runs, the reduction ranged between 3.1–5.9 log_10_, i.e. an elimination of > 99.9% of the BG spores. In the following eight runs, 112/114 (98.2%) respirator test pieces demonstrated ≥ 6 log_10_ reduction in BG viability and two test pieces remained just below this level. In the last two decontamination runs conducted in June 2020 with 12,000 and 20,000 respirators, BG spore viability was reduced by 3.4–4.4 log_10_, and 4.0–7.2 log_10_, respectively, indicating the elimination of > 99.96% of the spores. The 32 Spordex biological indicators used for these two large-scale runs all showed a pass result.

Each vertical bar depicts the range of reduction in the respirator test pieces distributed across various parts of the hydrogen peroxide vapour decontamination chamber, as compared with a concomitant control. The median reduction in *Bacillus atrophaeus* viability in each run is depicted by a horizontal line. The number of test pieces in the run is depicted below each bar.

The MS2 bacteriophage, used as a model virus only in the first two decontamination runs (21 respirator test pieces in each run) was also effectively destroyed by HPV, with a reduction in growth greater than 3.9 log_10_ pfu/sample observed.

### Particle penetration and breathing resistance

**Figure 4:**
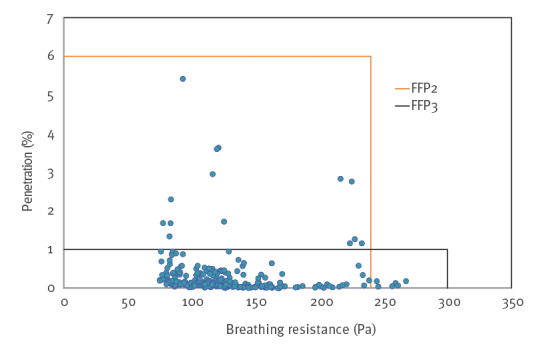
Particle penetration and breathing resistance measurements of randomly selected decontaminated respirators, Finland, April–June 2020

The particle penetration and breathing resistance of respirators that had been decontaminated once were at least the same as the requirements for class FFP2 respirators and mostly the same as requirements for class FFP3 respirators (Figure 4). The tolerance of elastic straps, tested by stretching, remained unchanged after repeated HPV exposures. The decontamination process, when repeated over 10 cycles, had no effect on the filtering efficacy of DEHS particles or on inhalation or exhalation resistance.

FFP2/FFP3: filtering facepiece respirator.

Measurement airflow rate 95 lpm as required by the EN 149 standard [18]. The standard penetration limits for FFP2 and FFP3 are shown in the figure.

No differences in filtering efficacy, airflow resistance and log reduction in the growth of decontamination indicator microbes were observed between respirators or test pieces placed in wire baskets in various ways among other respirators or at different locations in the chamber.

### Microbiological contamination of used respirators

The results of microbiological testing (Table 2) of used respirators collected from the participating hospitals demonstrated some microbial contamination before HPV treatment at the decontamination facility. However, there was no obvious difference in the contamination rate between respirators stored at + 4 °C or room temperature after varying storage times. The contaminating bacterial species consisted of commonly saprophytic skin microbes, such as catalase-producing *Staphylococcus saprophyticus*, *S. warneri* and *Micrococcus luteus*. Prior to HPV decontamination, the amount of bacterial growth exceeded 30 cfu/g of respirator in less than one fifth of respirators.

**Table 2:** Microbiological findings in used FFP2 and FFP3 respirators collected from nine healthcare districts, after various storage conditions before and after HPV treatment, Finland, April–June 2020 (n = 303)

Storage before microbiological testing		Samples tested (n)	Microbiological findings from respirators (cfu per 1 g of respirator)			
			< 30		≥ 30	
Duration (days)	Temperature		n	%	n	%
Prior to HPV decontamination treatment						
< 7	+ 4 °C	2	0	–	2	–
≥ 7	+ 4 °C	28	23	–	5	–
< 7	Ambient	2	1	–	1	–
≥ 7	Ambient	12	12	–	0	–
**Total**		**44**	**36**	**82**	**8**	**18**
After HPV decontamination treatment						
< 7	+ 4 °C	4	4	–	0	–
≥ 7	+ 4 °C	8	8	–	0	–
< 7	Ambient	235	235	100	0	–
≥ 7	Ambient	12	12	–	0	–
**Total**		**259**	**259**	**100**	**0**	**0**

cfu: colony-forming units; HPV: with hydrogen peroxide vapour; FFP2/FFP3: filtering facepiece respirator.

The EN 14683 standard defines 30 colony-forming units (cfu)/g as the maximum accepted microbiological contamination of unused respirators [17,18].

Percentages for totals under 40 are not shown.

After short-term storage of the decontaminated respirators, no microbial growth was found in two thirds of the tested respirators. In one third, small amounts of growth by saprophytic bacteria were observed, but the number of cfu per respirator (each weighing ca 10 g) was always below 300 cfu per respirator (threshold limit 30 cfu/g according to the SFS-EN 14683 standard [17]).

### Hydrogen peroxide concentration following decontamination and fit to face testing

During the decontamination process the respirators adsorbed hydrogen peroxide, which was gradually desorbed after the treatment. When measured with a dummy head, hydrogen peroxide concentration in the inhaled air dropped to < 0.1 ppm after 48 h.

Ten unused respirators of each model type from six models (n = 60) from two different manufacturers (3M: K113, 1833 +, K112, 9322, 9322 + ; GlaxoSmithKline: Antiviral Respirator Mask) were tested for fit to face before and after 20 HPV treatment runs. Differences in fit factors when tested before and after decontamination were not significant. All the measured over-all fit factors were higher than pass level 100. The lowest over-all fit factor of all tests was measured before HPV treatments: this was 133 for Antiviral Respirator Mask, which has only one headband. During donning, after 10 HPV treatment runs, a rubber band was detached from one Antiviral Respirator Mask and the respirator could not be retested.

## Discussion

In 12 weeks, we implemented a standardised collection system for used FFP2 and FFP3 respirators from hospitals responsible for the care of patients with severe acute respiratory syndrome coronavirus 2 (SARS-CoV-2) infections covering almost 90% of the 5.5 million population of Finland and constructed a facility for the large-scale decontamination of respirators using HPV. The process verified the efficacy of decontamination, as well as the preservation of the key filtering, breathing resistance and facial fit properties of the decontaminated respirators.

Based on the experience in producing HPV using a method developed by VTT and FDRA [15,16], our ad hoc group constructed a large-scale HPV decontamination facility on a large parking area using the FDF’s existing hardware. Scaling up the decontamination process from laboratory-based to large-scale processing necessitated high-capacity HPV generation and effective air circulation to ensure high and uniformly distributed HPV concentration inside a large space with densely packed respirators. Previously, the largest documented capacities for decontamination of respirators were up to 1,500 in one treatment run [21-25]. Our facility’s capacity to decontaminate 40,000 respirators per 24 h would meet the estimated maximum need for FFP2 and FFP3 respirators in Finland in the worst-case scenarios of the current pandemic, if no unused respirators were available.

After our initial decontamination runs, we observed high decontamination efficiency for the resistant *G. stearothermophilus* spores in the commercial biological indicators, as well as for the in-house indicator using BG spores, when the HPV concentration was over 350 ppm and the exposure was greater than 900 ppm*hours. This is in line with the high HPV decontamination efficacy reported previously from small-scale experiments, mostly conducted in the US [6,8,22,23,26]. In our two high-load runs of up to 20,000 respirators, which adsorb large amounts of HPV, we observed slightly lower reductions in the viability of BG spores deposited into the respirator matrix. This indicates > 99.96% reduction in BG viability even in these high load runs and, given the presence of pass results of the concomitant *G. stearothermophilus* spore commercial bioindicators, confirms high-level disinfection.

Bacterial spores are used to confirm success of disinfection. High-level disinfection means the destruction of all microorganisms except high numbers of bacterial spores [4]. Viruses, particularly lipid or medium-sized viruses, are clearly more susceptible to germicidal chemicals than bacterial spores [27]. In HPV decontamination studies on respirators, the MS2 phage we used, as well as the SARS-CoV-2 virus [9], the porcine respiratory coronavirus [28] and a range of other viruses [13,26], were effectively decontaminated.

In our study, the respirators retained their filtering efficacy and breathing resistance properties after 20 treatment cycles. In a Dutch investigation on decontamination with HPV, FFP2 respirators retained their form and met the criteria for filtering after two treatments [29]. In one study, the elastic material in the straps broke when extended after a large number of HPV treatments [8]. Our study demonstrated that the respirators treated with 20 cycles of HPV retained their fit in thorough testing of several types of respirators. Acceptable fit testing results after several cycles of HPV decontamination have been previously reported [9,14,24], but concerns have been raised about the integrity of fit after less than 20 HPV decontamination cycles for some respirator models [30,31].

The large-scale collection of respirators and decontamination in a large centralised facility offers several advantages in Finland, a country with a population of 5.5 million and long distances between the facilities involved. Foremost among them is that a centralised organisation can ensure the standardised quality of decontamination of respirators, in contrast to a decentralised system where a large number of healthcare facilities would use previously unfamiliar technology set up under substantial time pressure. With the technology used in our decontamination facility, the capacity could be doubled or tripled by adding similar units. Transportation logistics were not a substantial obstacle in spite of distances of up to 600 km from the most distant healthcare facilities that provided respirators to our project. In large countries, centralisation could mean regional or federal state-wide large-scale facilities.

One limitation of the study is that unused respirators were employed for fit tests to maximise the number of decontamination cycles. Additionally, test pieces of respirators contaminated with the in-house biological indicators were cut from unused respirators to simplify the testing procedures.

One of the lessons learned is that reducing the loss of respirators due to smearing by make-up products, making them unacceptable for reuse, requires intensive communication with healthcare staff. The objective was to provide the reprocessed respirators rapidly to healthcare districts, but this need never materialised. Therefore, the optimal conditions for long-term storage of decontaminated respirators were not investigated. The H_2_O_2_ concentration in the inhaled air using a dummy head was below one tenth of the occupational 8 h Finnish exposure limits, indicating that the use of the reprocessed respirators is safe. The facility has been dismantled, but it can be set up to operate at full capacity in 2 weeks if needed.

The reprocessed decontaminated respirators are likely to meet the technical test requirements of the EN 149 standard, but not all the standard and legislation requirements can be fulfilled. The respirators were tested for filtering efficacy, breathing resistance, fit to face, and headband elasticity. Testing of all the respirator properties according to the EN 149 standard was not considered essential because some properties are unlikely to change (e.g. carbon dioxide content of inhaled air), or are not considered to be important in healthcare use (e.g. flammability). We replaced the total inward leakage tests with fit tests with one person to acquire reproducible data on possible changes. The EU regulation prevents the use of reprocessed, decontaminated disposable respirators if the respirator does not have manufacturer instructions for decontamination [2], and they cannot be used in workplace [32]. Consequently, the objective of the investigation was not to provide reprocessed respirators to the healthcare districts or to the market while a sufficient supply of new ones was available. Given the availability of adequate supplies, the worst-case scenario that would have potentially required us to use the processed respirators did not materialise, and the decontaminated respirators were stored centrally. The European Centre for Disease Prevention and Control has published a review on various decontamination methods, including sterilisation in autoclave, HPV treatment, gamma radiation, ozone, UV and EtO treatment [33], without presenting decontamination efficacies for these methods or recommendations. In the US, the FDA has given an Emergency Use Authorization for use of reprocessed decontaminated respirators in the current pandemic, describing the conditions to be met in this process [3].

The WHO has raised the possibility of a serious, as yet unknown disease X [34] which could be transmitted in an airborne manner as effectively as, for example, measles, varicella and smallpox. In this case, if the general population were to require respirators in addition to healthcare, the demand for respirators would greatly surpass that experienced during the COVID-19 pandemic. This raises the need to consider using reprocessed respirators when unused ones are unavailable and to incorporate this concept in future preparedness processes.

Past pandemics have revealed national and international regulatory gaps or obstacles to harnessing societal resources to mitigate and control the impact of the pandemic. It is therefore important to systematically develop facilitated regulatory pathways for pandemics caused by serious diseases such as, for example, the airborne disease X scenario [35]. We propose that a regulatory pathway should be considered in the EU for the reuse of reprocessed respirators, decontaminated with HPV or other well-validated methods, as an emergency procedure when no new respirators are available, including strict conditions as a prerequisite for reuse.

## Conclusions

We have demonstrated the technical feasibility, decontamination efficacy and retention of key properties in large-scale decontamination of used respirators. The results indicate that the EU should consider, in the context of preparedness, the development of facilitated regulatory pathways to allow the reuse of respirators after well-defined decontamination processes, in a situation where new respirators are not available.

## Note

The datasets generated and/or analysed during the current study are not publicly available but are available from the corresponding author upon request.
